# Imbalanced learning: Improving classification of diabetic neuropathy from magnetic resonance imaging

**DOI:** 10.1371/journal.pone.0243907

**Published:** 2020-12-15

**Authors:** Kevin Teh, Paul Armitage, Solomon Tesfaye, Dinesh Selvarajah, Iain D. Wilkinson

**Affiliations:** 1 Academic Unit of Radiology, Department of Infection, Immunity and Cardiovascular Disease, University of Sheffield, Sheffield, United Kingdom; 2 Diabetes Research Department, Sheffield Teaching Hospitals NHS Foundation Trust, Sheffield, United Kingdom; 3 Department of Oncology and Metabolism, University of Sheffield, Sheffield, United Kingdom; Korea National University of Transportation, REPUBLIC OF KOREA

## Abstract

One of the fundamental challenges when dealing with medical imaging datasets is class imbalance. Class imbalance happens where an instance in the class of interest is relatively low, when compared to the rest of the data. This study aims to apply oversampling strategies in an attempt to balance the classes and improve classification performance. We evaluated four different classifiers from k-nearest neighbors (k-NN), support vector machine (SVM), multilayer perceptron (MLP) and decision trees (DT) with 73 oversampling strategies. In this work, we used imbalanced learning oversampling techniques to improve classification in datasets that are distinctively sparser and clustered. This work reports the best oversampling and classifier combinations and concludes that the usage of oversampling methods always outperforms no oversampling strategies hence improving the classification results.

## Introduction

Machine learning has enabled us to extract patterns from data to build predictive models. However, machine learning models tend to suffer from class imbalance especially in biomedical diagnosis [1]. In this context, class imbalance describes the skewed representation of a disease phenotype, whereby some classes appear more frequently [2]. Having an imbalanced class label can lead to biased learning classification in algorithms such as k-nearest neighbors (k-NN), support vector machines (SVM), decision trees (DT) and multilayer perceptron (MLP). This occurs as a result of inherent tendencies to preference and overfit towards the majority classes [3]. We assume in many machine learning classifier algorithms that the number of instances (classes) is roughly similar. However, by biasing training towards the majority classes, we risk overlooking the unique and occasionally more important minority classes.

**Fig 1:**
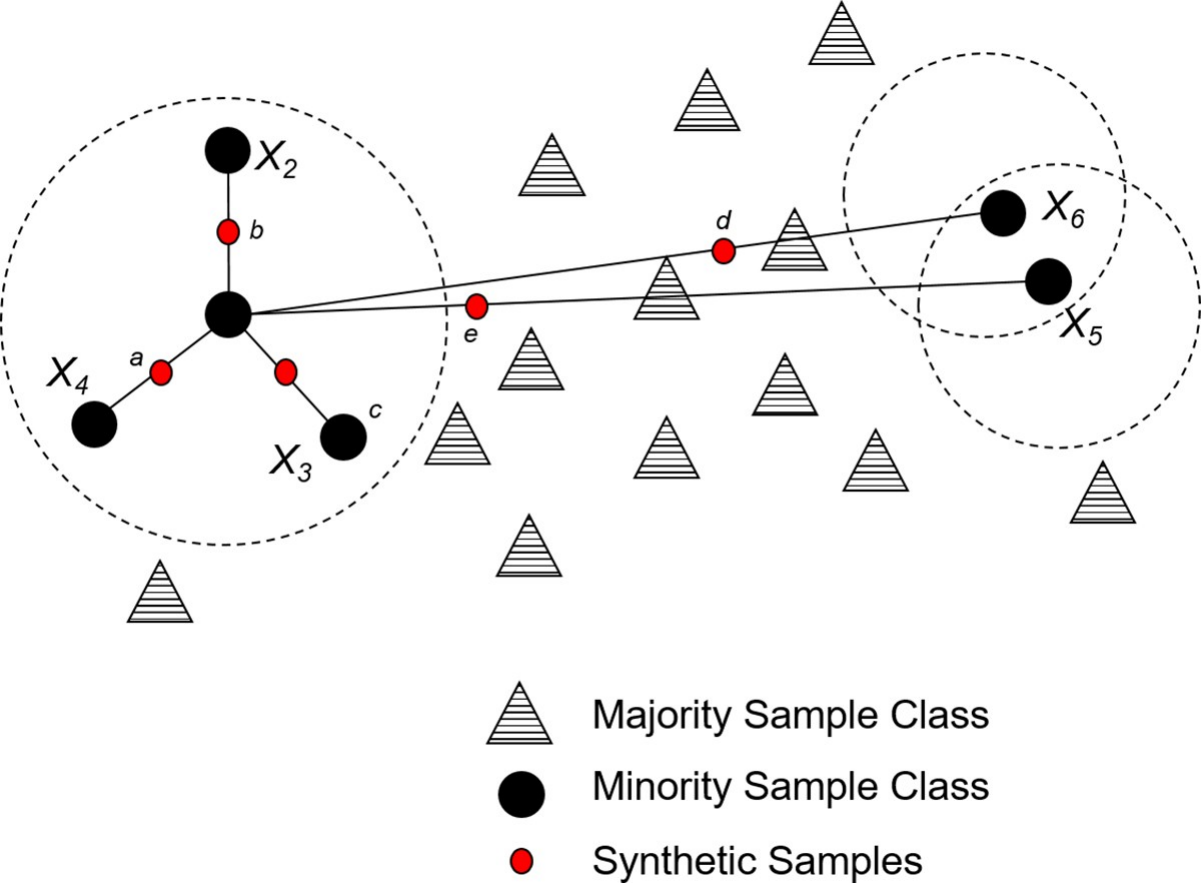
Depicts diagram of conventional SMOTE algorithm.

There are currently three categorical approaches to managing imbalanced data. The simplest method is to take an Algorithm level approach whereby classifiers are tuned for class imbalance based on existing classifier learning algorithms, one example is k-NN [4]. The second group of methods take a Data Level approach, which includes preprocessing methods (i.e the Synthetic Minority Oversampling Technique (SMOTE) [5]-see Fig 1), whereby additional training samples are generated for minority classes to rebalance the class distribution. The third method lies between the data and algorithm level approaches called the Cost Sensitive technique [6–8]. In these techniques, a higher cost is assigned to minority samples during the training process, well known examples are SVM[9] and ADACost [10]. In this paper, we only consider Data Level preprocessing methods, specifically oversampling methods. These methods tackle the root of the imbalanced learning problem, which is the lack of data. Secondly, they also allow easy application of a machine learning pipeline, unlike cost-sensitive and classifier specific solutions.

Some real life examples of class imbalanced problems include credit card fraud detection [11], text recognition [12] and crucially in healthcare diagnostics [13]. Increasingly advances in machine learning classification, especially in the field of medical imaging, are being used to diagnose diseases and predict treatment outcomes in various medical conditions [14]. In our work, we will be looking closely at classifying diabetic peripheral neuropathy (DPN) subjects. Diabetic peripheral neuropathy (DPN) is a common condition affecting half of all diabetic subjects and is a challenging condition to manage effectively [15]. With current treatments, the best outcome we can achieve is 50% pain relief in only a third of subjects. The current approach assumes that all subjects respond similarly to a given drug when in fact there is a wide variability in response. Over the last 10 years, we have demonstrated using magnetic resonance (MR) neuroimaging that altered brain structure and functional connectivity could serve as a possible Central Pain Signature (CPS) for painful DN [16, 17], which could provide a means of stratifying subjects to the right treatment first time.

**Fig 2:**
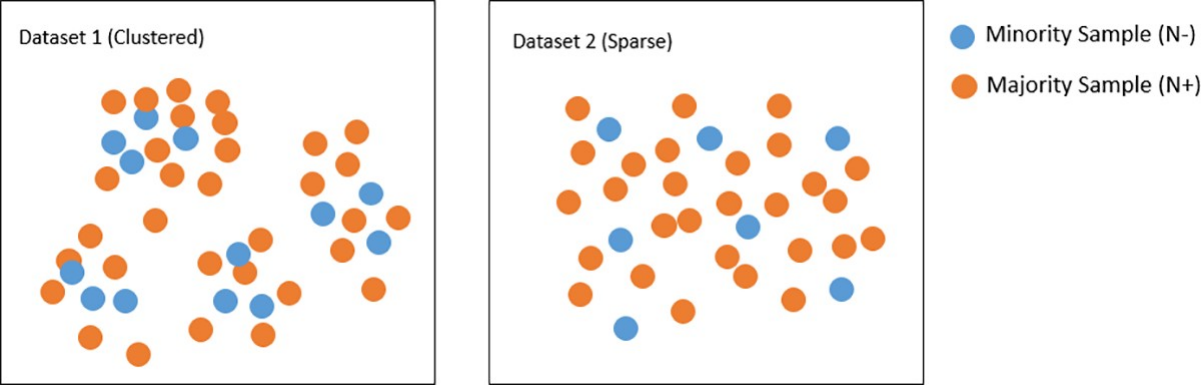
Depicts the two dataset described in Table 1. DTS1 above shows classes with more pockets of bunching together (Clustered) whereby the DTS2 is a more sporadic class dataset (Sparser).

The primary aim of this study was to determine whether oversampling improves the diagnostic performance of machine learning classification trained on MR imaging features. We compared 73 oversampling techniques against a baseline of no oversampling and conventional SMOTE to justify our exhaustive approach. Our secondary aim was to determine which oversampling strategies result in the best performance reported over two distinct datasets (clustered and sparse-see Fig 2). Both our datasets utilises MR imaging features specifically resting state and structural features commonly associated with the pain pathways in DPN subjects [16, 17]. The first consisted of an imbalanced binary classification dataset that is traditionally a multiclass classification problem. For this dataset, we tried to classify painful DPN from three other groups consisting of healthy volunteers (HV), no neuropathy (noDPN) and painless DPN. Our second sparser dataset investigated oversampling methods when applied to a smaller dataset in a particularly focused disease phenotype. Here we looked closer at painful DPN in particular responsiveness to treatment.

## Materials and methods

### MRI methods

#### Subjects

Our dataset comprises subjects with diabetes (n = 121) and heathy controls (n = 37). All subjects underwent detailed clinical and neurophysiological assessments to diagnose and phenotype DPN [18]. Subjects with diabetes were divided into three groups: no DPN(N = 42); painless DPN (N = 40) and painful DPN (N = 39). In the first analysis (DTS1), we classified subjects with painful DPN from the rest of the subjects. There were no significant differences in mean age or gender distribution (p > 0.05) between these two groups.

For the second analysis a different subset of subjects with painful DPN, which have been assessed for response to neuropathic pain treatment was used (DTS2). We divided these subjects into responders and non-responders. We used the NTSS-6 questionnaire, which grades neuropathic pain intensity and duration, to define responders [N = 13] with a score [19] below seven and non-responders [N = 40] with a pain score seven or above. Table 1 also shows the other characteristics of the two binary classification datasets used. Written informed consent for the study was obtained before subjects participated in the study which has prior approval by the Sheffield Local Research Ethics Committee.

**Table 1:** Shows parameters of the datasets used in this study.

Dataset	ATR	N(N+/N-)	IR
**DTS1**	14	158(119/39)	3.051
**DTS2**	13	53(40/13)	3.077

#### Dataset assessment

As shown in Table 1, Dataset 1 (DTS1-see S1 Table) comprises of diabetic and healthy control (HC) subjects separated into painful and non-painful subject classes. Dataset 2 (DTS2- see S2 Table) depicts only DPN subjects separated into responders and non-responders to treatment.

ATR refers to number of attributes or features used, column N is the total number of instances or subjects, N+ is the majority sample class, N- is the minority sample class and lastly IR is the imbalanced ratio (N+/N). Both datasets have similar imbalanced ratios with IR ≈ 3 and ATR number. Keeping IR and ATR constant allows us to focus this paper on exploring the two types of datasets. We kept the ATR similar by selecting the best features from our imaging data using recursive feature elimination (RFE) method as described in the next section. As shown in Fig 2, DTS1 have a more structured sub grouping or sub clustering structure as the non-painful class contains HC, painless and no DPN, which have distinctive neuroimaging characteristics. DTS2 however is a more random or sparser dataset with a smaller minority sample size. In addition, all the subjects in this dataset are painful DPN subjects making this dataset highly similar in neuroimaging characteristics.

#### Image acquisition & processing

*MRI acquisition*. In the weeks before treatment all subjects underwent MRI using a Phillips Achieva 3 Tesla system (Phillips Medical Systems, Holland) with a 32-channel head coil. Anatomical data were acquired using a T1-weighted magnetisation prepared rapid acquisition gradient echo sequence with the following parameters: repetition time (TR) 7.2 ms, echo time (TE) 3.2 ms, flip angle 8°, and voxel size 0.9 mm3, yielding isotropic spatial resolution. A 6-minute resting-state fMRI sequence was acquired while subjects fixated on a cross using a T2*-weighted pulse sequence, with TE = 35ms; TR = 2600ms, in-plane pixel dimensions = 1.8mmx1.8mm, contiguous trans-axial slices thickness of 4mm were orientated in the oblique axial plane parallel to the AC-PC bisection, covering the whole cerebral cortex.

*Resting state*. ROI-ROI based analysis was performed using the CONN (version 18.a) [20]: functional connectivity toolbox software. This software was also used to perform all preprocessing steps (using the default preprocessing pipeline), as well as subsequent statistical analyses, on all subject scans. In CONN’s preprocessing pipeline, raw functional images were slice-time corrected, realigned (motion corrected), unwarped, and coregistered to each subject's T1-weighted dataset in accordance with standard algorithms. Resulting images were then normalized to Montreal Neurological Institute (MNI) coordinate space, spatially smoothed (5 mm full-width at half maximum), and resliced to yield 2 × 2 × 2 mm voxels. Regional mean blood oxygenation level dependent time series were extracted from each patient for 10 chosen regions with each ROI defined with a spherical radius of 5mm. The 10 sources chosen were the insular cortex (l,r), postcentral gyrus(l,r), precentral gyrus(l,r), thalamus(l,r) and the cingulate gyrus(a,p) region. Pearson’s correlation coefficients were calculated for each region’s BOLD time series correlating with every other region’s BOLD time series to form a symmetric 10x164 matrix for each patient. The correlation coefficients were z-transformed using Fishers transform to normalize the distribution

*Structural processing*. Cortical reconstruction and volumetric segmentation were performed with FreeSurfer software [21] (http://surfer.nmr.mgh.harvard.edu). Preprocessing includes motion correction and averaging [22] of volumetric T1-weighted images, removal of non-brain tissue [23] using a hybrid watershed/ surface deformation procedure, affine registration to the Talairach atlas [24, 25], intensity normalization, tessellation of the gray matter-white matter boundary, automated topology correction [26, 27], and surface deformation following intensity gradients to optimally place the gray/white and gray/cerebrospinal fluid borders at the location where the greatest shift in intensity defines the transition to the other tissue class [28, 29]. Intensity and continuity information from the entire three-dimensional MR volume in segmentation and deformation procedures is used to produce surface-based maps. These maps subsequently produce representations of cortical thickness calculated as the closest distance from the gray/white to the gray/cerebrospinal fluid boundaries at each vertex on the tessellated surface (34). Cortical thickness (in mm), volumes of the insular cortex, postcentral gyrus, precentral gyrus, thalamus and the cingulate gyrus were assessed.

### Machine learning methods

#### Oversampling strategies

In this work we have kept the IR as one after the application of oversampling for all classification experiments. There are more than 100 variants of SMOTE in the literature [30], but we have only adopted 73 oversamplers in our study and have discounted techniques that are essentially similar. In total we conducted 292 oversampling classification experiments and four no oversampling experiments using four different classifiers. We have also categorised the oversamplers based on their key characteristic operating principles as reported by [31] and shown in S3 Table.

As shown in S3 Table, each oversampling method falls into a few operating principles, however some techniques are unique and does not fall into any particular operating principle. In the results section, we report which operating principles perform best on our two datasets. By reporting the best oversamplers in this way, it should also allow future studies with similarly sparser or clustered datasets to select potential oversamplers not described in this work. In the rest of this section, we will endeavor to summarise some of the most prevalent operating principles:

*Dimensionality reduction*. These techniques reduce the dimensions of the data to a lower dimensional space. Some common techniques are principal component analysis (PCA) and linear discriminant analysis (LDA).

*Component-wise sampling*. Attributes are sampled independently in this method and the assumption is that the entire volume of a hypercube spanned by two neighboring minority samples belongs to the minority class.

*Ordinary sampling*. These techniques are very similar to conventional SMOTE methods (see Fig 1) and adapt the underlying principle that new minority samples are generated in between two neighbouring minority line sections.

*Borderline*. Borderline methods increase the number of minority samples that border majority samples. The objective of using a borderline method is to allow the classifier to be able to distinguish between these borderline observations more easily.

*Using a sampling density*. The key principle of density based methods are to assign a weighted distribution for different minority class examples.

*Use of clustering*. In these methods, clustering techniques are used to identify minority concepts, and then the oversampling is done within the individual clusters independently.

#### Classifier algorithms

We used four different classifiers covering neural network methods, ensemble methods and lazy learners. These were chosen as they offer classifier diversity and have also been adopted most in imbalanced learning literature as base classifiers [32]. The four classifiers used were:

*k-NN*. K-nearest neighbor (k-NN) [33, 34] is a lazy learning algorithm storing all instances corresponding to training data points in n-dimensional space. Once new discrete data is received, it analyses the closest k number of instances saved (nearest neighbors) and returns the most common class as the prediction. We trained the k-NN classifier used in this work by optimising the number of nearest neighbours and using uniform weighting between them.

*SVM*. Linear support vector machines SVM [35] try to classify cases by finding a separating boundary termed hyperplane. The distances from the hyperplane boundary relate to the likelihood of a subject belonging to a class. SVM has been used in a range of problems and they have already been successful in pattern recognition in bioinformatics, cancer diagnosis [36] and other areas.

*MLP*. Multilayer perceptron is a class of feedforward deep, artificial neural network composing of more than three layers of nodes. They are the input layer, a hidden layer and an output layer whereby each input node is a neuron that uses a nonlinear activation function. In training MLP, a supervised learning technique called backpropagation is utilised.

*DT*. Decision tree uses a tree-like graph or model of decisions and their possible consequences and is an algorithm that only contains conditional control statements. This classifier uses the tree representation in which each leaf node corresponds to a class label and attributes are represented on the internal node of the tree.

#### Performance measures

One of the most crucial processes of defining a machine learning model is model evaluation. In binary classification problems traditional metrics such as accuracy, precision and recall have been widely accepted as standard evaluation measures. These are not suitable in imbalanced scenarios, since the performance of the majority class will be overrepresented. Usage of oversampling techniques maintains a reasonable performance for the majority samples whilst improving the classification of minority samples. We will compare three performance measures in this work. These are the G-score, F1 score and AUC score. Previous works have investigated the effectiveness of different measures and have concluded that these measures fit best for imbalanced data problems [37–40]. Firstly, introducing some acronyms TP, TN, FP and FN are the number of true positive, true negative, false positive and false negative samples, respectively, and P = TP +FN, N = TN +FP, the selected measures are defined as follows.

**G** score the geometric mean of accuracies achieved on minority and majority instance:

$$G=\sqrt{\frac{TP}{P}∙\frac{TN}{N}}$$ (1)

**F1** score is interpreted as a weighted average of the precision (PR) and recall (RE):

$$F1=2∙\frac{PR.RE}{PR+RE},$$ (2)

$$PR=\frac{TP}{TP+FP},$$ (3)

$$RE=\frac{TP}{TP+FN}$$ (4)

Where precision measures samples correctly classified as positive, and recall describes the proportion of all positive samples classified as positive.

**AUC** score (Area Under the receiver operating characteristic Curve) characterises the area under the curve of sensitivities plotted against corresponding false positive rates (FPR):

$$FPR=\frac{FP}{FP+TN}$$ (5)

#### Experimental protocol

**Fig 3:**
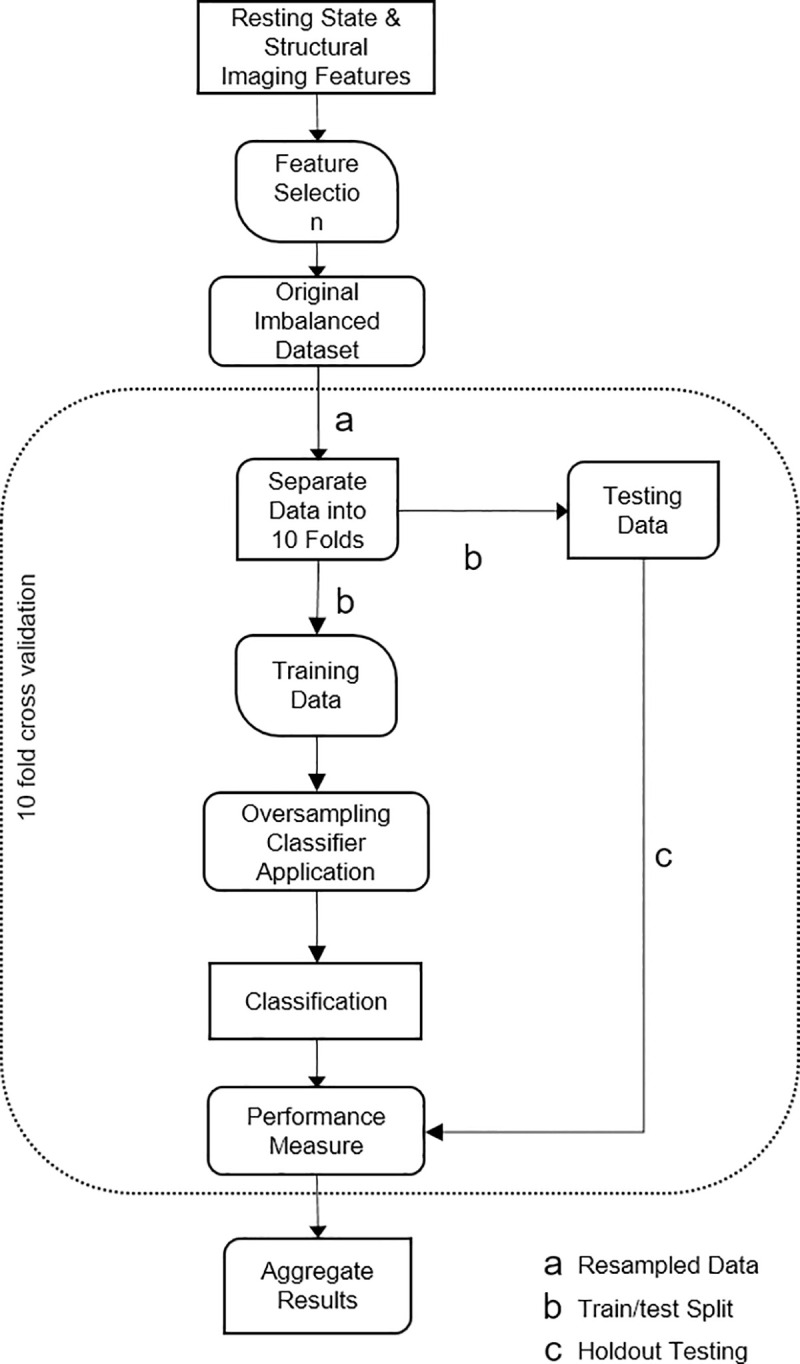
Schematic diagram illustrating the imbalanced learning workflow.

All analyses were performed using the Scikit-learn package in Python [41]. Oversampling algorithms based on S3 Table above were implemented by adapting a freely available imbalanced learning toolbox [42] to apply the described oversampling strategies. Our full experimental workflow can be seen in Fig 3 and is described below.

The first step in our experiment involved data exploration and cleaning. This involved selecting the most relevant regions (10 regions described in MRI Processing section above) from the resting state and volumetric brain image analysis in the brain that define our classification problems as reported in previous work [16, 17]. Next, both our datasets were numerically normalised and standardised to a common scale. A dimensionality reduction feature selection method was then implemented using the RFE method to avoid selecting highly correlated features. After feature selection the original imbalanced dataset was oversampled before cross validation. We also split the data with a 0.3/0.7 (training/testing split) split according to subject classes found in Table 1. We described in the rest of this subsection, the details of the evaluation methodology.

*Classifier parameters*. To evaluate each classifier we selected different combinations of hyperparameter tuning parameters. In our MLP classifier, we used one hidden layer and specified the logistic activation functions and hidden units as 10%, 50% and 100% of the number of input features. In our k-NN classifier, we used standard or distance weighted decision functions with L2 distance and the k voting neighbors as 3, 5 or 7. For the DT classifier, we selected Gini-impurity or entropy as the splitting criterion, unbounded and with a maximum depth of 3 and 5. We used a linear SVM with L1 and L2 penalties with compatible hinge or squared hinge loss and regularisation parameter C to be 1 and 10.

*Cross validation*. We evaluated classification performance by repeated stratified k-fold cross-validation with 10 splits and 10 repeats.

*Performance evaluation*. The performance of all oversampler classifier combination (OC) is carried out on 30 random oversampler parameter combination with six different classifier parameter combinations. We also oversampled the training set data before classifier training. We evaluated F1, AUC and G-score and reported the top results for each dataset, classifier and oversamplers. We consistently used an average score (average over AUC, F1 and G score) (AS) over the three performance measures in this paper to compare oversamplers and classifiers allowing an unbiased performance evaluation.

## Result

All detailed findings from 292 oversampling and four no oversampling experiments are shown in supplementary tables, S4 Table for DTS1 and S5 Table for DTS2.

### Oversampling algorithm comparison

We have shown the top 10 performers for each dataset in Tables 2 and 3 below, aggregated by the performance measure results over all four classifiers. Next, we ranked the oversamplers using the average rank (average AUC, F1 and G rank) to obtain a more unbiased oversampler ranking rather than ranking using AS.

**Table 2:** Top 10 performing oversamplers for DTS1 versus baseline (no oversampling and SMOTE) averaged across four classifiers.

DTS1									
Rank	sampler	AUC	F1	G	AS	AUCRank	F1Rank	GRank	avgRank
1	**Assembled_SMOTE**	0.7655	0.5603	0.7151	0.6803	3	3	5	3.67
2	**SL_graph_SMOTE**	0.7606	0.5635	0.7199	0.6813	12	1	1	4.67
3	**ProWSyn**	0.7629	0.5600	0.7143	0.6791	5	4	6	5.00
4	**polynom_fit_SMOTE**	0.7669	0.5585	0.7138	0.6797	2	10	7	6.33
5	**Lee**	0.7619	0.5592	0.7127	0.6779	7	7	9	7.67
6	**AND_SMOTE**	0.7629	0.5573	0.7120	0.6774	6	13	12	10.33
7	**CURE_SMOTE**	0.7670	0.5587	0.7095	0.6784	1	8	24	11.00
8	**Selected_SMOTE**	0.7633	0.5597	0.7094	0.6775	4	5	25	11.33
9	**SMOTE_FRST_2T**	0.7584	0.5563	0.7167	0.6771	17	17	2	12.00
10	**distance_SMOTE**	0.7602	0.5587	0.7103	0.6764	13	9	20	14.00
Baseline	**SMOTE**	0.7522	0.5436	0.7032	0.6663	49	46	41	45.33
Baseline	**No Oversampling**	0.6877	0.4041	0.5612	0.5510	72	74	74	73.33

**Table 3:** Top 10 performing oversamplers for DTS2 versus baseline (no oversampling and SMOTE) averaged across four classifiers.

DTS2									
Rank	sampler	AUC	F1	G	AS	AUCRank	F1Rank	GRank	avgRank
1	**Lee**	0.8903	0.7322	0.8501	0.8242	1	2	1	1.33
2	**polynom_fit_SMOTE**	0.8872	0.7337	0.8489	0.82327	3	1	3	2.33
3	**SMOTE_TomekLinks**	0.8854	0.7299	0.8494	0.82157	8	4	2	4.67
4	**SMOTE_IPF**	0.885	0.7305	0.8424	0.8193	9	3	7	6.33
5	**CE_SMOTE**	0.8894	0.7261	0.8388	0.8181	2	6	11	6.33
6	**Assembled_SMOTE**	0.8856	0.7239	0.8459	0.81847	7	9	4	6.67
7	**G_SMOTE**	0.8857	0.7226	0.8427	0.817	6	11	6	7.67
8	**SMOBD**	0.8839	0.7242	0.8452	0.81777	11	8	5	8
9	**Edge_Det_SMOTE**	0.8798	0.7258	0.8424	0.816	21	7	8	12
10	**SDSMOTE**	0.8823	0.7208	0.8409	0.81467	16	14	9	13
Baseline	**SMOTE**	0.8779	0.7086	0.8286	0.80503	22	28	29	26.33
Baseline	**No Oversampling**	0.831	0.5662	0.6795	0.69223	69	71	71	70.33

The top performers always perform better than baseline comparisons (no oversampling or SMOTE). Usage of oversampling measures, including baseline SMOTE, outperforms no oversampling measures as shown in Table 2. When compared to No Oversampling, the best oversampler (Rank 1) gives an improved AS performance of 12.9 percent for DTS1 and 13.2 percent for DTS2. We also conducted an independent samples T test to test the significance of oversampling versus no oversampling and reported a p value of 0.159 for DTS1 and 0.044 for DTS2.

Using baseline SMOTE also yields an AS boost of 14.2 percent for DTS1 and 11.3 percent for DTS2. Comparing baseline SMOTE to the best oversampler there was an improvement of 1.33 percent for DTS1 and 1.92 percent for DTS2. DTS1 also shows that Assembled_SMOTE [43] and SL_graph_SMOTE [44] are the best oversampling performers. Based on the average rank for DTS2 the Lee [45] and polynom-fit-SMOTE [46] oversampling algorithm performs the best.

### Classifier comparison

SVM is the best performing classifier compared to the other four classifiers (see Table 4). Based on AS scores, SVM performs 3.74 percent better than MLP the next best classifier for DTS1 and 5.42 percent better than MLP in DTS2. DT is also consistently the worst performing classifier choice. We also compared SVM versus the other 3 base classifiers when oversampling is used, this correlated to a p value of 0.024 for DTS1 and 0.044 for DTS2 when we conducted an independent T test.

**Table 4:** Shows the average and top performing AS over all oversamplers for the four different classifier types in DTS1 and DTS2.

DTS1				
Classifier	AUC	F1	G	AS
k-NN	0.7397	0.5225	0.6849	0.6490
MLP	0.7796	0.5597	0.7137	0.6843
DT	0.6597	0.4680	0.6369	0.5882
SVM	0.8170	0.6051	0.7430	0.7217
DTS2				
Classifier	AUC	F1	G	AS
k-NN	0.7948	0.8886	0.8130	0.8321
MLP	0.8124	0.9058	0.8300	0.8494
DT	0.6566	0.7116	0.7037	0.6907
SVM	0.8786	0.9462	0.8861	0.9037

### Oversampling classifier combination comparison

Top 10 best oversamplers with their respective classifiers is shown in Table 5. We ranked these based on AS scores and show that an oversampler combination with SVM classifier always outperforms oversampler combination with DT, k-NN and MLP. This is true for both the datasets. The SVM classifier provided the top performing AS score at 0.76 for DTS1 and 0.93 for DTS2. Looking into DTS1, the top performers using the SVM classifiers are A_SUWO [47] and Borderline_SMOTE1 [48], which perform the best amongst all the oversamplers. The top performing SVM oversampler combinations for DTS2 are SMOTE_Cosine [49] and Borderline_SMOTE1 [48] achieving the highest AS score.

**Table 5:** Shows the top performers ranked by AS scores over the four columns reporting the four classifier techniques used.

DTS1								
Classifier	SVM		DT		k-NN		MLP	
Rank	Sampler	AS	Sampler	AS	Sampler	AS	Sampler	AS
1	A_SUWO	0.7588	Borderline_SMOTE2	0.6253	CURE_SMOTE	0.6841	Stefanowski	0.7183
2	Borderline_SMOTE1	0.7563	MSMOTE	0.6169	polynom_fit_SMOTE	0.6836	polynom_fit_SMOTE	0.7153
3	SMOTE_ENN	0.7509	SMOTE_ENN	0.6144	NRAS	0.6831	SMOTE_D	0.7148
4	SL_graph_SMOTE	0.7499	SL_graph_SMOTE	0.6111	Gazzah	0.6814	CBSO	0.7136
5	Borderline_SMOTE2	0.7496	ISOMAP_Hybrid	0.6106	Gaussian_SMOTE	0.6786	DE_oversampling	0.7132
6	SMOTE_TomekLinks	0.747	AND_SMOTE	0.6103	ProWSyn	0.6777	MWMOTE	0.7132
7	SDSMOTE	0.7463	Assembled_SMOTE	0.6093	SOI_CJ	0.6766	distance_SMOTE	0.7095
8	SMOTE_FRST_2T	0.7436	ADOMS	0.6084	MDO	0.6749	ISMOTE	0.7091
9	LN_SMOTE	0.7431	LN_SMOTE	0.6083	Lee	0.6723	SN_SMOTE	0.7077
10	SMOBD	0.7417	SMOBD	0.6076	LLE_SMOTE	0.672	ADOMS	0.7055
**DTS2**								
Classifier	SVM		DT		k-NN		MLP	
Rank	Sampler	AS	Sampler	AS	Sampler	AS	Sampler	AS
1	SMOTE_Cosine	0.93	LVQ_SMOTE	0.7221	SMOTE_IPF	0.8406	Borderline_SMOTE2	0.8549
2	Borderline_SMOTE1	0.9263	Lee	0.7207	CE_SMOTE	0.838	cluster_SMOTE	0.8525
3	SDSMOTE	0.9203	SMOTE_D	0.7107	SMOTE_OUT	0.838	SMOTE_IPF	0.852
4	polynom_fit_SMOTE	0.92	SMOBD	0.7054	CBSO	0.8365	Edge_Det_SMOTE	0.85
5	G_SMOTE	0.9198	Assembled_SMOTE	0.7017	polynom_fit_SMOTE	0.835	SMOTE_FRST_2T	0.8494
6	SMOTE_OUT	0.9198	CE_SMOTE	0.6978	SMOTE_TomekLinks	0.8338	NDO_sampling	0.8477
7	Assembled_SMOTE	0.9196	G_SMOTE	0.6974	Selected_SMOTE	0.832	SMOTE_TomekLinks	0.8475
8	Lee	0.9188	NRSBoundary_SMOTE	0.6968	Borderline_SMOTE2	0.8284	CURE_SMOTE	0.8468
9	MWMOTE	0.9179	polynom_fit_SMOTE	0.6968	MWMOTE	0.8264	CBSO	0.8462
10	cluster_SMOTE	0.917	Random_SMOTE	0.696	CURE_SMOTE	0.8262	SMOTE_D	0.8449

Each row represents the oversampling technique providing the top results reported in descending order. We did this over both DTS1 and DTS2.

### Operating principles

The top three operating principles for DTS1 are Ordinary Sampling, Density Based and Application (see Table 6). Some examples of Ordinary Sampling are ProWSyn [50] and ADASYN [51]. ADASYN also falls in the Density Based category. Other examples of in the Density Based category are A_SUWO [47]. This oversampler is the top performing OC as shown in Table 5. The top three principles for DTS2 were Application, Uses Classifier and Ordinary Sampling. Examples of oversamplers in the Uses Classifier operating principle includes SMOTE_IPF [52]. The next top principle was Application, these were oversamplers developed for specific applications with an example of CE_SMOTE [53] oversampler. CE_SMOTE and SMOTE_FRST_2T [54] also falls in both the Application and Ordinary Sampling principle which is placed in two of the top three operating principles for DTS2. On the contrary, we observed moderate performance when using density estimation or dimensionality reduction.

**Table 6:** Table comparing operating principles over DTS1 and DTS2 based upon oversamplers categorized in S3 Table.

	DTS1		DTS2	
RANK	Operating Principle	AS	Operating Principle	AS
1	Ordinary Sampling	0.6689	Application	0.8004
2	Density Based	0.6677	Uses Classifier	0.8002
3	Application	0.6656	Ordinary Sampling	0.7949
4	Uses Clustering	0.6644	Uses Clustering	0.7940
5	Componentwise Sampling	0.6626	Componentwise Sampling	0.7928
6	Borderline	0.6624	Density based	0.7884
7	Uses Classifier	0.6614	Borderline	0.7861
8	Memetic	0.6597	Sampling By Cloning	0.7807
9	Dimensionality Reduction	0.6563	Changes Majority	0.7624
10	Changes Majority	0.6549	Noise Removal	0.7614
11	Noise Removal	0.6505	Memetic	0.7600
12	Sampling By Cloning	0.6489	Dimensionality Reduction	0.7506
13	Density Estimation	0.6383	Density Estimation	0.7310

## Discussion

The two key findings from this study were 1) using an oversampling strategy results in better classification performance than not oversampling at all. When we implemented oversampling on average across both datasets and performance measures, we demonstrated a 14.3 percent improvement. 2) classification can be improved further by empirically using 73 oversamplers. We reported the best oversamplers, classifier and operating principles for DTS1 and DTS2. In this work, we did not review or discuss oversampling techniques in depth, however objectively using these as a measure for classification improvement. We have also not considered undersampling or hybrid methods as we have only have a small minority total sample size.

For DTS1 (more clustered), Assembled SMOTE and SL_graph_SMOTE were the best oversamplers. Both these techniques are categorised principally as Borderline methods. These methods perform well on DTS1 as the clusters of minority and majority samples are more assembled together or closely packed. Hence, by using Borderline methods we can distinguish between the two instances more easily. The top 5 oversamplers for DTS 1 are further discussed in S1 Appendix. Individually the best overall OC is A_SUWO when used with SVM. This method is ideal for this dataset as it works best to differentiate sub-clusters of minority samples from majority classes that are close together. It oversamples the sub-clusters by assigning weights to their instances whilst avoiding generating overlapping synthetic instances by considering the majority instances that overlap minority ones. In terms of operating principles, Ordinary Sampling, Density Based and Application methods are the best performers. These principles are ones that implement oversampling very similar to conventional SMOTE. One reason these are successful is that it makes the right compromise between introducing variance and staying close to the original distribution of our dataset.

In DTS2, a smaller and sparser dataset with a smaller minority sample. The best overall oversampler was Lee whereby a noise filtering approach very similar to the k-NN approach was used. Using a post-processing noise-filtering step enhances the performance on a small minority sampled dataset. The top 5 oversamplers for DTS 2 are further discussed in S1 Appendix. Individually the best OC is SMOTE-Cosine with SVM as the base classifier. This oversampler has been shown to work better with the SVM classifier [49]. Oversampling based on Application and Uses Classifier operating principles gives the best performance, whereas the worst performance is achieved with density estimation and dimensionality reduction. These methods fail due to the number of minority samples being extremely low (N- = 13) and the number of attributes (ATR = 13) is not smaller than the number of N-. Secondly due to the sparse nature of this dataset N- can sometimes be mistakenly identified as noise [31].

In terms of number of misclassified cases, there was little change when comparing the oversampling techniques for both DTS1 and DTS2. However, we observed a significant change in misclassification as compared to no oversampling. When we used oversampling in DTS1, we found a misclassification of 21.1 percent or 15 out of 71 misclassified cases as compared to 27.6 percent or 13 out of 47 cases in the no oversampling case. When we used oversampling, we found a similar trend for DTS2 with 8.3 percent misclassification or 2 out of 24 cases as compared to 12.5 percent or 2 of 16 cases when no oversampling was considered. We reported these numbers based on a 70/30 training testing split and using the top OC performer for both datasets.

As a whole, SVM performs better than the other three classifiers. SVM is robust, precise and easier to train on smaller datasets. SVM also has the ability to generate nonlinear decision boundaries using methods designed for linear classifiers and adopts a flexible decision boundary. This adaptive boundary ability is very important in handling the problem of imbalanced datasets [9, 55]. In our results, we have found the AUC scores are comparatively higher as compared to the F1 or G score. This was because the features (ATR) were selected maximising AUC. There is scope to improve the AS scores further by better feature selection and hyperparameter tuning of the classifier (in the present study, parameter tuning was constrained by computational time). However, we emphasise that the objective of this work was primarily to compare oversamplers over our two datasets rather than pushing AS scores. Future experiments will explore a larger sample size for the smaller datatset (DTS2). We will also explore using other base classifiers such as unsupervised principle component analysis and K-means clustering techniques.

To conclude, we have reported the most appropriate oversampling approaches for two distinct datasets. In addition, to our knowledge, this is the first study addressing 73 different oversampling strategies to improve the diagnostic performance of machine learning classification on MRI datasets. Our findings provide an insight into the best approach to improving the binary classification of imbalanced datasets.

## Supporting information

S1 Table DTS1 feature dataset. (XLSX)

S2 Table DTS2 feature dataset. (XLSX)

S3 Table Principle operating characteristics of oversampling strategies used. (DOCX)

S4 Table Detailed results from 296 classification experiments for DTS1. (XLSX)

S5 Table Detailed results from 296 classification experiments for DTS2. (XLSX)

S1 Appendix Extended summary of top 5 oversamplers for DTS1 and DTS 2. (DOCX)
