# Supplementary material for: Imbalanced learning: Improving classification of diabetic neuropathy from magnetic resonance imaging
Source: PLoS One. 2020 Dec 15;15(12):e0243907. doi: 10.1371/journal.pone.0243907 (PMC7737960; doi:10.1371/journal.pone.0243907)
Supplement: S3 Table — (DOCX) [file pone.0243907.s003.docx]

**S3 Table. Principle operating characteristics of oversampling strategies used [1].**

| **Oversampling Method** | Noise Removal | Dimensionality Reduction | Uses Classifier | Componentwise Sampling | Ordinary Sampling | Sampling By Cloning | Memetic | Density Estimation | Density Based | Changes Majority | Uses Clustering | Borderline | Application |
| --- | --- | --- | --- | --- | --- | --- | --- | --- | --- | --- | --- | --- | --- |
| ADASYN[2] |  |  |  |  | x |  |  |  | x |  |  | x |  |
| ADOMS[3] |  | x |  |  |  |  |  |  |  |  |  |  |  |
| AHC[4] |  |  |  |  |  |  |  |  |  | x | x |  | x |
| AND_SMOTE[5] |  |  |  |  | x |  |  |  |  |  |  |  |  |
| ANS[6] |  |  |  |  | x |  |  |  |  | x |  |  |  |
| ASMOBD[7] | x |  |  |  |  |  |  |  |  |  | x |  |  |
| A_SUWO[8] | x |  |  |  |  |  |  |  | x |  | x |  |  |
| Assembled_SMOTE[9] |  |  |  |  | x |  |  |  |  |  | x | x |  |
| Borderline_SMOTE1[10] |  |  |  |  | x |  |  |  |  |  |  | x |  |
| Borderline_SMOTE2[10] |  |  |  |  | x |  |  |  |  |  |  | x |  |
| CBSO[11] |  |  |  |  | x |  |  | x |  |  | x |  |  |
| CCR[12] |  |  |  |  |  |  |  |  |  |  |  |  |  |
| CE_SMOTE[13] |  |  |  |  | x |  |  |  |  |  |  | x | x |
| CURE_SMOTE[14] |  |  |  |  |  |  |  |  |  |  | x |  |  |
| DBSMOTE[15] |  |  |  |  |  |  |  |  |  | x |  |  |  |
| DE_oversampling[16] |  |  |  |  |  |  |  |  |  | x | x |  |  |
| E_SMOTE[17] |  | x |  |  |  |  | x |  | x | x |  | x |  |
| Edge_Det_SMOTE[18] |  |  |  |  |  |  |  |  | x |  |  | x |  |
| GASMOTE[19] |  |  |  |  | x |  | x |  |  |  |  |  |  |
| G_SMOTE[20] |  |  |  | x |  |  |  |  |  |  |  |  |  |
| Gaussian_SMOTE[21] |  |  |  |  |  |  |  |  |  |  |  |  |  |
| Gazzah[22] |  | x |  |  |  |  |  |  |  | x |  |  |  |
| ISMOTE[23] |  |  |  |  |  |  |  |  | x |  |  |  |  |
| ISOMAP_Hybrid[24] | x | x |  |  |  |  |  |  |  | x |  |  |  |
| KernelADASYN[3] |  |  |  |  |  |  |  | x |  |  |  | x |  |
| LLE_SMOTE[25] |  | x |  |  |  |  |  |  |  |  |  |  |  |
| LN_SMOTE[26] |  |  |  | x |  |  |  |  |  |  |  |  |  |
| LVQ_SMOTE[27] |  |  |  |  |  |  |  |  |  |  |  |  | x |
| Lee[28] |  |  |  |  | x |  |  |  |  |  |  |  |  |
| MCT[29] |  |  |  |  |  | x |  |  |  |  |  |  |  |
| MDO[30] |  | x |  |  |  |  |  |  |  |  |  |  |  |
| MSMOTE[31] | x |  |  |  |  |  |  |  |  |  |  | x |  |
| MSYN[32] |  |  |  |  |  |  |  |  |  | x |  |  |  |
| MWMOTE[33] |  |  |  |  |  |  |  |  |  |  | x | x |  |
| NDO_sampling[34] |  |  |  |  | x |  |  |  |  |  |  |  |  |
| NRAS[35] | x |  |  |  | x |  |  |  |  |  |  |  |  |
| NRSBoundary_SMOTE[36] |  |  |  |  |  |  |  |  |  |  |  | x |  |
| NT_SMOTE[37] |  |  |  |  |  |  |  |  |  |  |  |  | x |
| OUPS[35] |  |  |  |  | x |  |  |  |  |  |  |  |  |
| PDFOS[38] |  |  |  |  |  |  |  | x |  |  |  |  |  |
| ProWSyn[39] |  |  |  |  | x |  |  |  |  |  |  |  |  |
| ROSE[40] |  |  |  | x |  |  |  |  |  |  |  |  |  |
| RWO_sampling[41] |  |  |  |  |  |  |  |  |  |  |  |  |  |
| Random_SMOTE[42] |  |  |  | x |  |  |  |  |  |  |  |  |  |
| SDSMOTE[43] |  |  |  |  | x |  |  |  |  |  |  | x |  |
| SL_graph_SMOTE[44] |  |  |  |  |  |  |  |  |  |  |  | x |  |
| SMMO[45] |  |  | x |  |  |  |  |  |  |  |  | x |  |
| SMOBD[46] | x |  |  |  |  |  |  |  | x |  | x |  |  |
| SMOTE[47] |  |  |  |  | x |  |  |  |  | x |  |  |  |
| SMOTE_Cosine[48] |  |  |  |  |  |  |  |  |  |  |  |  |  |
| SMOTE_D[49] |  |  |  |  |  |  |  |  |  |  |  |  |  |
| SMOTE_ENN[50] | x |  |  |  | x |  |  |  |  | x |  |  |  |
| SMOTE_FRST_2T[51] | x |  |  |  | x |  |  |  |  | x |  |  | x |
| SMOTE_IPF[52] |  |  | x |  |  |  |  |  |  | x |  |  |  |
| SMOTE_OUT[48] |  |  |  |  |  |  |  |  |  |  |  |  |  |
| SMOTE_RSB[53] |  |  |  |  | x |  |  |  |  |  |  |  |  |
| SMOTE_TomekLinks[50] | x |  |  |  | x |  |  |  |  | x |  |  |  |
| SN_SMOTE[54] |  |  |  |  | x |  |  |  |  |  |  |  |  |
| SOI_CJ[55] |  |  |  | x |  |  |  |  |  |  | x |  |  |
| SOMO[56] |  |  |  |  |  |  |  |  |  |  | x |  |  |
| SPY[57] |  |  |  |  |  |  |  |  |  | x |  |  |  |
| SUNDO[58] |  |  |  |  |  |  |  |  |  | x |  |  |  |
| SVM_balance[59] |  |  | x |  |  |  |  |  |  | x |  |  |  |
| Safe_Level_SMOTE[60] |  |  |  | x |  |  |  |  |  |  |  | x |  |
| Selected_SMOTE[48] |  |  |  | x |  |  |  |  |  |  |  |  |  |
| Stefanowski[61] | x |  |  |  |  | x |  |  |  | x |  | x |  |
| TRIM_SMOTE[62] |  |  |  |  |  |  |  |  |  |  | x |  |  |
| VIS_RST[63] | x |  |  |  |  |  |  |  |  | x |  |  |  |
| V_SYNTH[64] |  |  |  |  | x |  |  |  |  |  | x |  |  |
| cluster_SMOTE[65] |  |  |  |  |  |  |  |  |  |  | x |  |  |
| distance_SMOTE[45] |  |  |  |  | x |  |  |  |  |  |  |  |  |
| kmeans_SMOTE[66] |  |  |  |  |  |  |  |  |  |  | x |  |  |
| polynom_fit_SMOTE[67] |  |  |  |  |  |  |  |  |  |  |  |  |  |

References

1. Kovács G. Smote-variants: A python implementation of 85 minority oversampling techniques. Neurocomputing. 2019.

2. He HB, Bai Y, Garcia EA, Li ST. ADASYN: Adaptive Synthetic Sampling Approach for Imbalanced Learning. 2008 Ieee International Joint Conference on Neural Networks, Vols 1-8. 2008:1322-8.

3. Tang S, Chen S-p, editors. The generation mechanism of synthetic minority class examples. 2008 International Conference on Information Technology and Applications in Biomedicine; 2008: IEEE.

4. Cohen G, Hilario M, Sax H, Hugonnet S, Geissbuhler A. Learning from imbalanced data in surveillance of nosocomial infection. Artificial intelligence in medicine. 2006;37(1):7-18.

5. Yun J, Ha J, Lee J-S, editors. Automatic determination of neighborhood size in SMOTE. Proceedings of the 10th International Conference on Ubiquitous Information Management and Communication; 2016: ACM.

6. Siriseriwan W, Sinapiromsaran K. Adaptive neighbor synthetic minority over-sampling technique under 1NN outcast handling. Songklanakarin J Sci Technol. 2017;39:565-76.

7. Wang S, Li Z, Chao W, Cao Q, editors. Applying adaptive over-sampling technique based on data density and cost-sensitive SVM to imbalanced learning. The 2012 International Joint Conference on Neural Networks (IJCNN); 2012: IEEE.

8. Nekooeimehr I, Lai-Yuen SK. Adaptive semi-unsupervised weighted oversampling (A-SUWO) for imbalanced datasets. Expert Syst Appl. 2016;46:405-16.

9. Zhou B, Yang C, Guo H, Hu J, editors. A quasi-linear SVM combined with assembled SMOTE for imbalanced data classification. The 2013 International Joint Conference on Neural Networks (IJCNN); 2013: IEEE.

10. Han H, Wang W-Y, Mao B-H, editors. Borderline-SMOTE: a new over-sampling method in imbalanced data sets learning. International conference on intelligent computing; 2005: Springer.

11. Barua S, Islam MM, Murase K, editors. A novel synthetic minority oversampling technique for imbalanced data set learning. International Conference on Neural Information Processing; 2011: Springer.

12. Koziarski M, Wożniak M. CCR: A combined cleaning and resampling algorithm for imbalanced data classification. International Journal of Applied Mathematics and Computer Science. 2017;27(4):727-36.

13. Chen S, Guo G, Chen L, editors. A new over-sampling method based on cluster ensembles. 2010 IEEE 24th International Conference on Advanced Information Networking and Applications Workshops; 2010: IEEE.

14. Ma L, Fan S. CURE-SMOTE algorithm and hybrid algorithm for feature selection and parameter optimization based on random forests. BMC bioinformatics. 2017;18(1):169.

15. Bunkhumpornpat C, Sinapiromsaran K, Lursinsap C. DBSMOTE: density-based synthetic minority over-sampling technique. Applied Intelligence. 2012;36(3):664-84.

16. Chen L, Cai Z, Chen L, Gu Q, editors. A novel differential evolution-clustering hybrid resampling algorithm on imbalanced datasets. 2010 Third International Conference on Knowledge Discovery and Data Mining; 2010: IEEE.

17. Deepa T, Punithavalli M, editors. An E-SMOTE technique for feature selection in high-dimensional imbalanced dataset. 2011 3rd International Conference on Electronics Computer Technology; 2011: IEEE.

18. Kang Y-I, Won S, editors. Weight decision algorithm for oversampling technique on class-imbalanced learning. ICCAS 2010; 2010: IEEE.

19. Jiang K, Lu J, Xia K. A novel algorithm for imbalance data classification based on genetic algorithm improved SMOTE. Arabian journal for science and engineering. 2016;41(8):3255-66.

20. Sandhan T, Choi JY, editors. Handling imbalanced datasets by partially guided hybrid sampling for pattern recognition. 2014 22nd International Conference on Pattern Recognition; 2014: IEEE.

21. Lee H, Kim J, Kim S. Gaussian-Based SMOTE Algorithm for Solving Skewed Class Distributions. International Journal of Fuzzy Logic and Intelligent Systems. 2017;17(4):229-34.

22. Gazzah S, Hechkel A, Amara NEB, editors. A hybrid sampling method for imbalanced data. 2015 IEEE 12th International Multi-Conference on Systems, Signals & Devices (SSD15); 2015: IEEE.

23. Li H, Zou P, Wang X, Xia R, editors. A new combination sampling method for imbalanced data. Proceedings of 2013 Chinese Intelligent Automation Conference; 2013: Springer.

24. Gu Q, Cai Z, Zhu L, editors. Classification of imbalanced data sets by using the hybrid re-sampling algorithm based on isomap. International Symposium on Intelligence Computation and Applications; 2009: Springer.

25. Wang JJ, Xu MT, Wang H, Zhang JW. Classification of imbalanced data by using the SMOTE algorithm and locally linear embedding. Int Conf Sign Proces. 2006:1815-+.

26. Maciejewski T, Stefanowski J, editors. Local neighbourhood extension of SMOTE for mining imbalanced data. 2011 IEEE Symposium on Computational Intelligence and Data Mining (CIDM); 2011: IEEE.

27. Nakamura M, Kajiwara Y, Otsuka A, Kimura H. Lvq-smote–learning vector quantization based synthetic minority over–sampling technique for biomedical data. BioData mining. 2013;6(1):16.

28. Lee J, Kim N-r, Lee J-H, editors. An over-sampling technique with rejection for imbalanced class learning. Proceedings of the 9th International Conference on Ubiquitous Information Management and Communication; 2015: ACM.

29. Jiang L, Qiu C, Li C. A novel minority cloning technique for cost-sensitive learning. International Journal of Pattern Recognition and Artificial Intelligence. 2015;29(04):1551004.

30. Abdi L, Hashemi S. To combat multi-class imbalanced problems by means of over-sampling techniques. Ieee T Knowl Data En. 2015;28(1):238-51.

31. Hu S, Liang Y, Ma L, He Y, editors. MSMOTE: improving classification performance when training data is imbalanced. 2009 second international workshop on computer science and engineering; 2009: IEEE.

32. Fan X, Tang K, Weise T, editors. Margin-based over-sampling method for learning from imbalanced datasets. Pacific-Asia Conference on Knowledge Discovery and Data Mining; 2011: Springer.

33. Barua S, Islam MM, Yao X, Murase K. MWMOTE--majority weighted minority oversampling technique for imbalanced data set learning. Ieee T Knowl Data En. 2012;26(2):405-25.

34. Zhang L, Wang W, editors. A re-sampling method for class imbalance learning with credit data. 2011 International Conference of Information Technology, Computer Engineering and Management Sciences; 2011: IEEE.

35. Rivera WA, Xanthopoulos P. A priori synthetic over-sampling methods for increasing classification sensitivity in imbalanced data sets. Expert Syst Appl. 2016;66:124-35.

36. Hu F, Li H. A novel boundary oversampling algorithm based on neighborhood rough set model: NRSBoundary-SMOTE. Mathematical Problems in Engineering. 2013;2013.

37. Xu YH, Li H, Le LP, Tian XY, editors. Neighborhood triangular synthetic minority over-sampling technique for imbalanced prediction on small samples of chinese tourism and hospitality firms. 2014 Seventh International Joint Conference on Computational Sciences and Optimization; 2014: IEEE.

38. Gao M, Hong X, Chen S, Harris CJ, Khalaf E. PDFOS: PDF estimation based over-sampling for imbalanced two-class problems. Neurocomputing. 2014;138:248-59.

39. Barua S, Islam MM, Murase K, editors. ProWSyn: Proximity weighted synthetic oversampling technique for imbalanced data set learning. Pacific-Asia Conference on Knowledge Discovery and Data Mining; 2013: Springer.

40. Menardi G, Torelli N. Training and assessing classification rules with imbalanced data. Data Mining and Knowledge Discovery. 2014;28(1):92-122.

41. Zhang H, Li M. RWO-Sampling: A random walk over-sampling approach to imbalanced data classification. Information Fusion. 2014;20:99-116.

42. Dong Y, Wang X, editors. A new over-sampling approach: random-SMOTE for learning from imbalanced data sets. International Conference on Knowledge Science, Engineering and Management; 2011: Springer.

43. Li K, Zhang W, Lu Q, Fang X, editors. An improved SMOTE imbalanced data classification method based on support degree. 2014 International Conference on Identification, Information and Knowledge in the Internet of Things; 2014: IEEE.

44. Bunkhumpornpat C, Subpaiboonkit S, editors. Safe level graph for synthetic minority over-sampling techniques. 2013 13th International Symposium on Communications and Information Technologies (ISCIT); 2013: IEEE.

45. De La Calleja J, Fuentes O, González J, editors. Selecting Minority Examples from Misclassified Data for Over-Sampling. FLAIRS Conference; 2008.

46. Gao M, Hong X, Chen S, Harris CJ, editors. Probability density function estimation based over-sampling for imbalanced two-class problems. The 2012 international joint conference on neural networks (IJCNN); 2012: IEEE.

47. Chawla NV, Bowyer KW, Hall LO, Kegelmeyer WP. SMOTE: synthetic minority over-sampling technique. J Artif Intell Res. 2002;16:321-57.

48. Koto F, editor SMOTE-Out, SMOTE-Cosine, and Selected-SMOTE: An enhancement strategy to handle imbalance in data level. 2014 International Conference on Advanced Computer Science and Information System; 2014: IEEE.

49. Torres FR, Carrasco-Ochoa JA, Martínez-Trinidad JF, editors. SMOTE-D a deterministic version of SMOTE. Mexican Conference on Pattern Recognition; 2016: Springer.

50. Batista GE, Prati RC, Monard MC. A study of the behavior of several methods for balancing machine learning training data. ACM SIGKDD explorations newsletter. 2004;6(1):20-9.

51. Ramentol E, Gondres I, Lajes S, Bello R, Caballero Y, Cornelis C, et al. Fuzzy-rough imbalanced learning for the diagnosis of High Voltage Circuit Breaker maintenance: The SMOTE-FRST-2T algorithm. Engineering Applications of Artificial Intelligence. 2016;48:134-9.

52. Sáez JA, Luengo J, Stefanowski J, Herrera F. SMOTE–IPF: Addressing the noisy and borderline examples problem in imbalanced classification by a re-sampling method with filtering. Information Sciences. 2015;291:184-203.

53. Ramentol E, Caballero Y, Bello R, Herrera F. SMOTE-RSB*: a hybrid preprocessing approach based on oversampling and undersampling for high imbalanced data-sets using SMOTE and rough sets theory. Knowledge and information systems. 2012;33(2):245-65.

54. García V, Sánchez JS, Martín-Félez R, Mollineda RA. Surrounding neighborhood-based SMOTE for learning from imbalanced data sets. Progress in Artificial Intelligence. 2012;1(4):347-62.

55. Sanchez AI, Morales EF, Gonzalez JA. Synthetic oversampling of instances using clustering. International Journal on Artificial Intelligence Tools. 2013;22(02):1350008.

56. Douzas G, Bacao F. Self-Organizing Map Oversampling (SOMO) for imbalanced data set learning. Expert Syst Appl. 2017;82:40-52.

57. Dang XT, Tran DH, Hirose O, Satou K, editors. SPY: A novel resampling method for improving classification performance in imbalanced data. 2015 Seventh International Conference on Knowledge and Systems Engineering (KSE); 2015: IEEE.

58. Cateni S, Colla V, Vannucci M, editors. Novel resampling method for the classification of imbalanced datasets for industrial and other real-world problems. 2011 11th International Conference on Intelligent Systems Design and Applications; 2011: IEEE.

59. Farquad M, Bose I. Preprocessing unbalanced data using support vector machine. Decision Support Systems. 2012;53(1):226-33.

60. Bunkhumpornpat C, Sinapiromsaran K, Lursinsap C, editors. Safe-level-smote: Safe-level-synthetic minority over-sampling technique for handling the class imbalanced problem. Pacific-Asia conference on knowledge discovery and data mining; 2009: Springer.

61. Stefanowski J, Wilk S, editors. Selective pre-processing of imbalanced data for improving classification performance. International Conference on Data Warehousing and Knowledge Discovery; 2008: Springer.

62. Puntumapon K, Waiyamai K, editors. A pruning-based approach for searching precise and generalized region for synthetic minority over-sampling. Pacific-Asia Conference on Knowledge Discovery and Data Mining; 2012: Springer.

63. Borowska K, Stepaniuk J, editors. Imbalanced data classification: A novel re-sampling approach combining versatile improved SMOTE and rough sets. IFIP International Conference on Computer Information Systems and Industrial Management; 2016: Springer.

64. Young WA, Nykl SL, Weckman GR, Chelberg DM. Using Voronoi diagrams to improve classification performances when modeling imbalanced datasets. Neural Computing and Applications. 2015;26(5):1041-54.

65. Cieslak DA, Chawla NV, Striegel A, editors. Combating imbalance in network intrusion datasets. GrC; 2006.

66. Douzas G, Bacao F, Last F. Improving imbalanced learning through a heuristic oversampling method based on k-means and SMOTE. Information Sciences. 2018;465:1-20.

67. Gazzah S, Amara NEB, editors. New oversampling approaches based on polynomial fitting for imbalanced data sets. 2008 The Eighth IAPR International Workshop on Document Analysis Systems; 2008: IEEE.
