# Supplementary material for: Imbalanced learning: Improving classification of diabetic neuropathy from magnetic resonance imaging
Source: PLoS One. 2020 Dec 15;15(12):e0243907. doi: 10.1371/journal.pone.0243907 (PMC7737960; doi:10.1371/journal.pone.0243907)
Supplement: S1 Appendix — (DOCX) [file pone.0243907.s006.docx]

**DTS 1 top 5 performers**

**Assembled_SMOTE [1]**

Compared to conventional SMOTE assembled SMOTE implements oversampling by considering the data distribution information of the border minority class samples hence avoiding occurrence of overlapping between classes. Firstly, local linear partitions are obtained each of which can be separated with one linear separation boundary. Next using the information of local linear partitions the assembled SMOTE generates synthetic minority class samples. The final step involves using quasi-linear SVM to realise a classification of oversampled datasets in the same way as a standard SVM by using a composite quasi-linear kernel function.

**SL_graph_SMOTE [2]**

This oversampling method is based upon the concept of a safe level graph is to describe the characteristics of the minority class’s instances based on the k nearest neighbors as shown by the safe level oversampling method [3]. Firstly, a safe level graph is plotted representing the dataset distribution via graph skewness either left or right. This skewness is then used to choose between the usage of two SMOTE oversampling methods namely Safe Level SMOTE and Borderline SMOTE [4]. This method is a great choice when datasets have a clearly defined safe or borderline minority instances as described by [3, 4].

**ProWSyn [5]**

This technique results in a proper distribution of generated synthetic samples across the minority data set. This is done by generating effective weight values for the minority data samples based on sample’s proximity information (i.e., distance from boundary). ProWSyn is unique among the sampling density techniques because the algorithm generates new instances by sampling the line segments between minority instances having similar distances to majority instances. ProWSyn is useful when minority instances are relatively far from one another.

**Lee [6]**

The Lee technique generates synthetic data by considering its location. There is a rejecting method associated with this technique if the generated synthetic data is considered as noise data. A rejection level is assigned to each synthetically generated instance defined as how many positive instances are located with its 5 nearest neighbors. This technique is great when the imbalanced ration is high, the authors demonstrated the techniques on imbalanced ratio of (1:5- 1:20) with good results.

**polynom_fit_SMOTE [7]**

This technique refers to 4 fairly different oversampling strategies controlled by the topology parameter of the technique. The topologies used are the ’bus’, ’star’, ’mesh’ and ’polynom’. Using this technique generates instances along line segments between relatively far samples of the minority class. Hence like ProWSyn this techniques is most useful when the minority class is more scattered.

**DTS 2 top 5 performers**

The Lee and polynom_fit_SMOTE technique has already been discussed above

**SMOTE_TomekLinks [8]**

These methods combines Smote with a data cleaning methods namely Tomek links. This is one of the first extensions of SMOTE motivated by its well known drawback of generating overlapped and noisy examples. SMOTE method is firstly applied to oversample the minority class to a balanced distribution, then examples in Tomek Links from the majority classes are identified and removed. Pairs of nearest neighbors in a dataset that have different classes are identified via Tomek links and by removing one or both of the examples in these pairs (such as the examples in the majority class) has the effect of making the decision boundary in the training dataset less noisy or ambiguous.

**SMOTE_IPF [9]**

SMOTE-IPF is a noise filtering oversampling algorithm, which can overcome the problems produced by noisy and borderline examples in imbalanced datasets. Firstly, it executes ordinary SMOTE to generate new minority samples, then uses a supervised classifier with cross-validation to check the consistency of new samples and remove the ones with low probabilities of belonging to the minority class. This technique iteratively eliminates noisy samples. Samples removed in one iteration do not influence detection in subsequent ones, resulting in a more accurate noise filtering. This makes SMOTE-IPF more robust than that of Borderline-SMOTE[4] and SL-SMOTE [3] as it discards the neighborhood calculation. However, SMOTE-IPF fails to assign the individual parameters for different instances by considering their distribution characteristics.

**CE_SMOTE [10]**

CE-SMOTE applies cluster ensembles technology to effectively find out the boundary minority samples to be over-sampled, and thus improving the performance of a classifier for both minority and majority classes. This technique seeks out boundary minority samples (i.e. the minority samples which frequently change their cluster assignments in multi-partitions) and then over-sample these boundary minority samples to balance the original data set. CE-SMOTE only over-samples the boundary minority samples which are found out by cluster ensembles.

# **References**

1. Zhou B, Yang C, Guo H, Hu J, editors. A quasi-linear SVM combined with assembled SMOTE for imbalanced data classification. The 2013 International Joint Conference on Neural Networks (IJCNN); 2013: IEEE.

2. Bunkhumpornpat C, Subpaiboonkit S, editors. Safe level graph for synthetic minority over-sampling techniques. 2013 13th International Symposium on Communications and Information Technologies (ISCIT); 2013: IEEE.

3. Bunkhumpornpat C, Sinapiromsaran K, Lursinsap C, editors. Safe-level-smote: Safe-level-synthetic minority over-sampling technique for handling the class imbalanced problem. Pacific-Asia conference on knowledge discovery and data mining; 2009: Springer.

4. Han H, Wang W-Y, Mao B-H, editors. Borderline-SMOTE: a new over-sampling method in imbalanced data sets learning. International conference on intelligent computing; 2005: Springer.

5. Barua S, Islam MM, Murase K, editors. ProWSyn: Proximity weighted synthetic oversampling technique for imbalanced data set learning. Pacific-Asia Conference on Knowledge Discovery and Data Mining; 2013: Springer.

6. Lee J, Kim N-r, Lee J-H, editors. An over-sampling technique with rejection for imbalanced class learning. Proceedings of the 9th International Conference on Ubiquitous Information Management and Communication; 2015.

7. Gazzah S, Amara NEB, editors. New oversampling approaches based on polynomial fitting for imbalanced data sets. 2008 The Eighth IAPR International Workshop on Document Analysis Systems; 2008: IEEE.

8. Batista GE, Prati RC, Monard MC. A study of the behavior of several methods for balancing machine learning training data. ACM SIGKDD explorations newsletter. 2004;6(1):20-9.

9. Saez J, Luengo J, Stefanowski J, Herrera F. Addressing the noisy and borderline examples problem in classification with imbalanced datasets via a class noise filtering method-based re-sampling technique. Inform Sci. 2015;291:184-203.

10. Chen S, Guo G, Chen L, editors. A new over-sampling method based on cluster ensembles. 2010 IEEE 24th International Conference on Advanced Information Networking and Applications Workshops; 2010: IEEE.
